# Copper-Free *‘Click’* Chemistry-Based Synthesis and Characterization of Carbonic Anhydrase-IX Anchored Albumin-Paclitaxel Nanoparticles for Targeting Tumor Hypoxia

**DOI:** 10.3390/ijms19030838

**Published:** 2018-03-13

**Authors:** Katyayani Tatiparti, Samaresh Sau, Kaustubh A. Gawde, Arun K. Iyer

**Affiliations:** 1 Use-Inspired Biomaterials & Integrated Nano Delivery (U-BiND) Systems Laboratory, Department of Pharmaceutical Sciences, Eugene Applebaum College of Pharmacy and Health Sciences, Wayne State University, Detroit, MI 48201, USA; katyayani.tatiparti@wayne.edu (K.T.); kaustubhagawde@gmail.com (K.A.G.); 2 Molecular Imaging Program, Barbara Ann Karmanos Cancer Institute, Wayne State University School of Medicine, Detroit, MI 48201, USA

**Keywords:** carbonic anhydrase IX, tumor hypoxia targeting, paclitaxel, copper free ‘*click*’ chemistry, triple negative breast cancer, albumin nanoparticles, human serum albumin

## Abstract

Triple negative breast cancer (TNBC) is a difficult to treat disease due to the absence of the three unique receptors estrogen, progesterone and herceptin-2 (HER-2). To improve the current therapy and overcome the resistance of TNBC, there is unmet need to develop an effective targeted therapy. In this regard, one of the logical and economical approaches is to develop a tumor hypoxia-targeting drug formulation platform for selective delivery of payload to the drug-resistant and invasive cell population of TNBC tumors. Toward this, we developed a Carbonic Anhydrase IX (CA IX) receptor targeting human serum albumin (HSA) carriers to deliver the potent anticancer drug, Paclitaxel (PTX). We used Acetazolamide (ATZ), a small molecule ligand of CA IX to selectively deliver HSA-PTX in TNBC cells. A novel method of synthesis involving copper free ‘*click*’ chemistry (Dibenzocyclooctyl, DBCO) moiety with an azide-labeled reaction partner, known as Strain-Promoted Alkyne Azide Cycloaddition (SPAAC) along with a desolvation method for PTX loading were used in the present study to arrive at the CA IX selective nano-carriers, HSA-PTX-ATZ. The anticancer effect of HSA-PTX-ATZ is higher compared to HSA, PTX and non-targeted HSA-PTX in MDA-MB-231 and MDA-MB-468 cells. The cell killing effect is associated with induction of early and late phases of apoptosis. Overall, our proof-of-concept study shows a promising avenue for hypoxia-targeted drug delivery that can be adapted to several types of cancers.

## 1. Introduction

Cancer is a one the major causes of death in the U.S.A., annually claiming more than half a million lives and an estimated 1.5 million new cases [1]. Thus, there is an urgent need to improve diagnostic tools for early detection and to develop more selective drug delivery agents for therapy of cancer with least toxic side effects. Nanomedicines have been shown to be multitasking drug delivery vehicles that can passively accumulate within tumor tissue and have been clinically approved for conventional cancer therapy. Accumulated evidence indicates that current drug delivery agents failed in clinical trials due to lack of targeting ability, poor tumor penetration, tumor heterogeneity, and complex association of tumor associated immune cells and stroma [2]. Thus, targeted drug delivery that utilizes various ligands to recognize specific biomarkers expressed on tumor components has become extremely important in selective delivery of drugs and enhancing therapeutic efficacy [3,4,5,6,7,8,9,10,11,12,13,14,15,16]. The targeted cancer treatment approach differentiates between healthy and cancer tissues [17,18]. One smart way of designing a successful approach for tumor targeting can be the development of tumor multi-component targeting ligand library, and formulations with various types of biocompatible drug-carriers. This library of ligands could be surface decorated with drug-carrier systems using a combinatorial reagent-free organic synthesis approach for improving tumor early diagnosis and therapy. Modern research methods have the potential to transform personalized cancer treatment with improved quality of life, while providing the opportunity to integrate chemistry, drug delivery, cancer research elements and discoveries into multidisciplinary research experiences. The research presented in this study is based on a similar idea, i.e.,

(i) self-assembling of polymer, lipid, metallic-based nanosized drug-carrier, and bio-manufacturing of endogenous cell- (red blood cell, exosome) and protein (serum albumin, transferrin)-based cargo carrier [3,6,8,19,20,21,22,23,24,25,26,27,28,29,30,31,32,33];
(ii) functionalization of targeting ligands with the cargo carriers using a reagent-free synthesis approach, such as copper free cyclic Alkyne-Azide click reaction, “Thiol-Ene” Michael-type, and strain-promoted “Alkyne-Nitrone” cycloadditions [34,35,36,37,38,39,40,41,42,43].

The beauty of all these reactions is that they do not require any harmful chemicals, reagents, or any special condition, and they yield highly specific coupled products. The new knowledge gained through this interdisciplinary effort will provide a unique repertoire of advancement in drug delivery with rational design of targeting ligand-customized cargo carriers to exert distinct functions in the tumor microenvironment. The present study is an attempt to apply this simple strategy to develop a formulation of human serum albumin (HSA) via the copper-free cyclic Alkyne-Azide click reaction that targets tumor hypoxia.

Furthermore, nanoparticles have been broadly utilized as drug delivery systems for targeted delivery of anticancer drugs [44,45,46,47,48,49,50,51,52,53,54,55,56,57,58,59,60,61,62,63,64] in the pharmaceutical industry. They can enhance the delivery of hydrophobic drugs, decrease metabolic degradation of these drugs, target their delivery to malignant cancer cells just by the alteration of the surface of the delivery system with the addition of a ligand, and show controlled, extended or sustained delivery of drugs [7,8,13,25,53,58,65,66,67,68,69,70,71,72,73,74,75,76,77,78,79,80,81]. Alongside the capacity to improve the solubility of hydrophobic drugs, nanomicelles additionally can target cancer cells by two different strategies: passive and active targeting. The passive targeting delivery systems rely on the capacity of nanomicelles to exploit the Enhanced Permeability and Retention (EPR) effect [61,82,83,84,85,86,87,88]. The EPR phenomenon proposes that the multiplication of cancer cells brings about the improvement of profoundly disordered and flawed veins. Hence, nanoparticles including nanomicelles can extravasate and gather at the tumor site [89,90]. Hence, the nanomicelles are commonly conjugated with a targeting moiety in the targeted delivery system, thus, encouraging the specific aggregation of the drugs in target tissues, singular cancer cells, or intracellular organelles that are related to specific targeting biomarkers in cancer cells [83,84]. However, the presence of the EPR effect is not common to all cancer cells and, in some, may not be as effective as in others [86,91]. Further, in those cancer cells that do show the EPR effect, angiogenesis is not uniform across the tumor depth which can cause unequal distribution of the drugs. This gives a reason to develop targeted delivery systems that function via active uptake by the cancer cells via receptor-mediated cell uptake [92]. The present study is one such attempt.

Albumin is the most inexhaustible plasma protein in the human body. Its anti-immunogenic properties make albumin a favored component as a sole carrier or a component of a carrier system for an assortment of cancer medications. Like the vast majority of plasma proteins, albumin is produced in the liver where it is created at a rate of around 0.7 mg/h for each gram of liver (i.e., 10–15 g daily). The normal half-life of albumin is 19 days. Its high take-up in inflamed and tumor cells makes it a perfect carrier for medications to target malignant cells. Numerous research- and industry-based techniques have been mentioned for in the literature, which conjugate or encapsulate different medications to albumin. Human serum albumin protein has a total set of 585 amino acids. A solitary polypeptide chain comprises 17 disulfide bonds, one free thiol and one tryptophan functional groups which make it an effective binding protein [93]. The advantages of the human serum albumin (HSA) include the presence of several drug binding sites like the amino and carboxylic groups that provide opportunities for covalent modifications and drug or protein attachment of the drugs or targeting ligands [94,95,96]. It has low-cost, non-immunogenic and naturally biodegradable properties that have been applied as a matrix for nanoparticles-based drug delivery systems [97,98,99]. The most important property that makes it preferable as a drug delivery carrier may be the fact that it is not susceptible to opsonization by serum proteins [100,101]. HSA conjugation could provide nanoparticles that are a promising delivery system for biopharmaceutically challenging medications, for example Paclitaxel (PTX), and they enhance the medication delivery and bioavailability for such drugs [102,103].

Carbonic Anhydrase (CA) is a zinc metalloenzyme that converts CO_2_ to bicarbonate reversibly generating a proton in the process. There are about 15 varieties of CAs in mammals each of which exhibit different properties, but all of them are involved in ion exchange and pH balance [104]. Of all of them, CA IX is the most efficient and is available on the surface of cancer cells. The enzyme’s active site is exposed outside the cell membrane. It is not expressed in normal healthy cells but is overexpressed in cancer cells under the influence of hypoxia [80,105], that is in turn produced by the extensively and fast-growing tumor cells which outgrow the oxygen supply creating a scenario of hypoxia. Thus, it has become the primary target for cancer therapy [106,107,108]. Sulfonamides are a group of anti-bacterial agents that have been recently proven to have antitumor activity too [109,110]. Acetazolamide (ATZ) is an aromatic member of the family of sulfonamides that is different from the other members of the group in that it does not have antibacterial properties. It was used for glaucoma and epilepsy but has also been proven to have carbonic anhydrase inhibitory activity. It has been used as an antitumor agent for 40 years. Other aromatic sulfonamides have also been found to have antitumor activities [111,112].

Paclitaxel is an effective anticancer drug used for chemotherapy. It has a unique chemical structure and results in polymerization of tubulin to stabilize microtubules and, furthermore, associates specifically with microtubules, making them stable against depolymerization in the presence of calcium and at cold temperatures, which otherwise promptly depolymerize ordinary microtubules. It is also known to act against mitochondria and inhibit apoptosis inhibitor protein B-cell Leukemia 2 (Bcl-2) [113,114,115,116,117]. However, it is highly hydrophobic and needs to be delivered via a delivery system that improves its solubility and delivers it at the target sites [16].

The research described in this article is one such attempt to design a targeted delivery system to deliver Paclitaxel to triple negative breast cancer (TNBC) cancer cell lines and test its efficacy in vitro. The results show a promising avenue for targeted delivery that can be adapted to several types of cancers.

## 2. Results

### 2.1. Synthesis of the Hypoxia Targeting Drug Delivery System

The product was prepared as described in Section 4.1. The final formulation was dialyzed and lyophilized to form a powder. The resultant product was then analyzed by FTIR (Fourier-transform infrared spectroscopy) to confirm the presence of the components used and the formation of the product from click reaction. The results of FTIR (Figure S1) showed characteristic peaks between 1700–2100 cm^−1^ corresponding to the ^−^N== ^+^N== ^−^N (azide) stretching and there is an observable shift in the peaks that indicates that the reaction has occurred. It also shows peaks for –N–H, –C=O, and –C–H bond stretching at 3300–3500, 1670–1820, and 1050–1350 cm^−1^ respectively. These results were further confirmed by ^1^H NMR spectrum (Figure S2) that show peaks between 6.8–7.4 ppm that correspond to the hydrogens of the Dibenzocyclooctyl (DBCO) and between 2.2–3.6 ppm indicating the hydrogens of the azide apart from the peaks for the sulfonamides between 7.8–8.0 ppm.

### 2.2. Drug Loading

The process of drug loading was performed at room temperature as described in the synthesis in Section 4.1. The extent of drug loading was computed utilizing High performance liquid chromatography (HPLC) and UV Spectrophotometry at 227 nm for absorbance of PTX. The concentration of drug loaded was computed by utilizing a standard graph previously established. The drug loading was observed to be around 11.3% *w*/*w* for PTX in this hypoxia-targeting drug delivery system.

### 2.3. Particle Size Analysis

**Figure 1:**
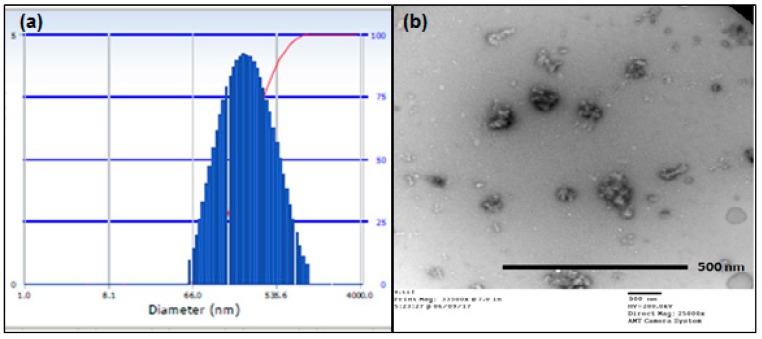
(**a**) Particle size by Dynamic light scattering (DLS) of the hypoxia targeting drug delivery system HSA-PTX-ATZ; (**b**) Particle size and morphology by Transmission electron microscopy (TEM) of the hypoxia targeting drug delivery system HSA-PTX-ATZ.

The investigations for measurement of the particle size of the formulation of this study were performed by the Dynamic light scattering (DLS) and Transmission electron microscopy (TEM). The outcomes demonstrated that the particle size (Figure 1a) on average was 294.0 nm and the polydispersity index (PDI) was around 0.139 demonstrating that the vast majority of the particles were inside the scope of this normal size. Further, the molecule size and morphology were considered utilizing TEM which indicated particles (Figure 1b) of the size between 105 and 130 nm. Both these outcomes affirm that the particle size is within the nanoscale.

### 2.4. Drug Release Studies

**Figure 2:**
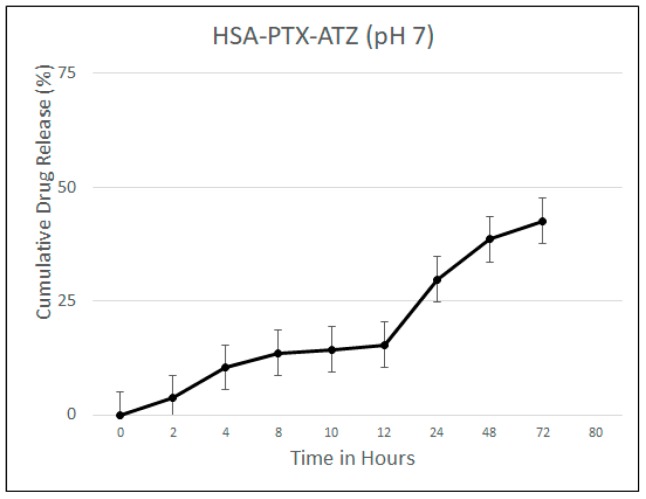
Drug release studies at pH 7 for the hypoxia targeting drug delivery system HSA-PTX-ATZ.

Drug release studies were carried out as described in Section 4.4. The drug release studies were carried out at the physiological pH of 7 because it corresponds to that of the blood. The drug released after the albumin nanoparticles were broken down was calculated using both UV and HPLC and the results were consistent. The release was found to be steady over a period of 72 h with a release of about 29.75% at 24 h and 42.62% at 72 h (Figure 2). This shows that the product under study can be considered as a sustained release formulation.

### 2.5. Stability Studies

**Figure 3:**
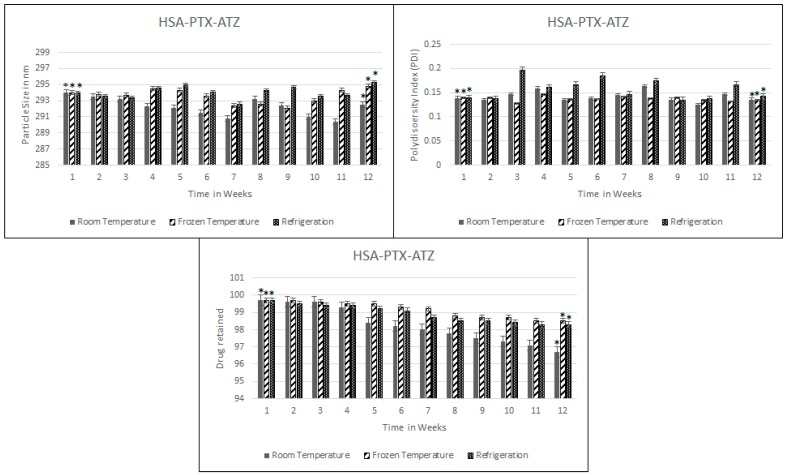
Stability studies over a period of 12 weeks for the hypoxia targeting drug delivery system HSA-PTX-ATZ. * Statistically not significant.

Stability studies are preformed to test the resistance of the formulation to drug loss over a period of time and to the aggregation of nanoparticles. Thus, these studies have been done in three aspects of particle size, PDI and the drug loss over a period of 12 weeks or three months and the results are shown in Figure 3. As can be seen, the particle size seems to very slightly fluctuate but after a slight amount of agitation, the particle size seemed to be more or less the same; 292.5 nm at room temperature. The particle size was found to be more stable when frozen over that time than at the other two conditions. Similarly, there have been fluctuations in the PDI and at the end of the 12 weeks it was around 0.136. As for the drug loss, the loss was at 3.0% at room temperature after 12 weeks but was lesser in the frozen conditions which was of the order of 1.4%.

### 2.6. In Vitro Cytotoxicity Studies

**Figure 4:**
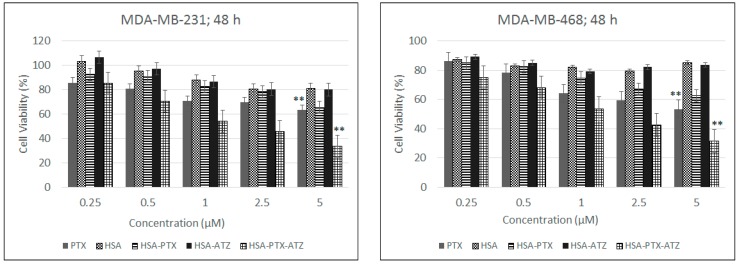
In vitro cytotoxicity studies in MDA-MB-231 and MDA-MB-468 cell lines respectively for the hypoxia targeting drug delivery system HSA-PTX-ATZ. ** Statistically significant.

These studies were performed on two different cell lines each expressing the CA IX receptor to a different extent [111,112]. In this direction, the results (Figure 4) of the in vitro cytotoxicity studies have shown that the MDA-MB-231 shows an IC_50_ at around 1 μm of the drug in the formulation as well as 1 μm in the MDA-MB-468 cell line as calculated via graphical and mathematical calculations from both MS Excel and GraphPad Prism 7. The results showed a dose-dependent killing and also a cell viability much lower than the non-targeted preparation and free drug in both cell lines at each concentration. The higher viability of the cells of the order of 80–90% for the carrier ligand combination additionally shows that the carrier along with ligand are safe for use. Statistical analysis was performed to calculate the significance of difference between free PTX and HAS-PTX-ATZ formulation in the two cell lines.

### 2.7. Comparative In Vitro Cytotoxicity Studies for Normoxic and Hypoxic Conditions

**Figure 5:**
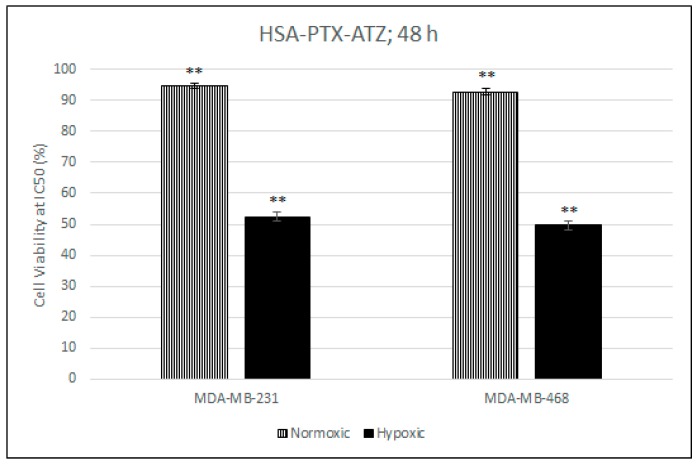
Comparative in vitro cytotoxicity studies for normoxic and hypoxic conditions in MDA-MB-231 and MDA-MB-468 cell lines respectively for the hypoxia targeting drug delivery system HSA-PTX-ATZ. ** Statistically significant.

This test proves that the presence of the ligand results in a higher uptake of the formulation in the hypoxic conditions and also that the CA IX receptor expression in normoxic conditions is much lower than the hypoxic conditions which results in the difference in uptake of the formulation in hypoxic conditions. The hypoxic condition was induced by the use of CoCl_2_ [118,119]. The normoxic condition was achieved by the absence of treatment of the cells with CoCl_2_. This was carried out at the IC_50_ concentration for the formulation in both cell lines calculated from the in vitro cytotoxicity studies. The results from Figure 5 show that the cytotoxicity of the formulation in the hypoxic conditions is much less than in the hypoxic conditions with the values of the cell viability being about 92% in normoxic conditions and around 51% in the hypoxic condition in both cell lines. This also proves that the formulation is quite safe in normal cells.

### 2.8. Fluorescence Spectroscopic Studies

**Figure 6:**
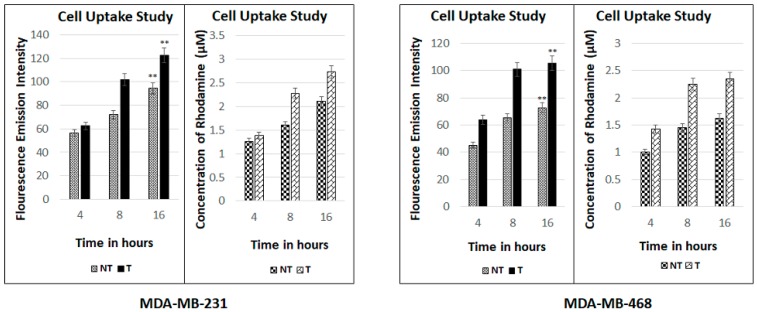
Fluorescence spectroscopic studies in MDA-MB-231 and MDA-MB-468 cell lines respectively for the hypoxia targeting drug delivery system HSA-PTX-ATZ. ** Statistically significant.

The fluorescence spectroscopic assay determines quantitatively the amount of drug entering the cells. The conjugation of rhodamine B to the drug and the nanomicelles and its subsequent detection in the spectrometer is time- and drug-concentration-dependent. The more rhodamine concentration detected with time means the more of the formulation along with the encapsulated drug might be entering the cells. The results of this assay show that the uptake of the formulation in the cells of both the cell lines increases with time taken at the time intervals of 4, 8, 16 h (Figure 6). The results have been presented in terms of fluorescence emission intensity as well as the concentration of rhodamine B that is conjugated to the formulation. The graph also depicts that the uptake of the targeted formulation in both the cell lines is more than the non-targeted formulation.

### 2.9. Apoptosis Assay by Flow Cytometry

**Figure 7:**
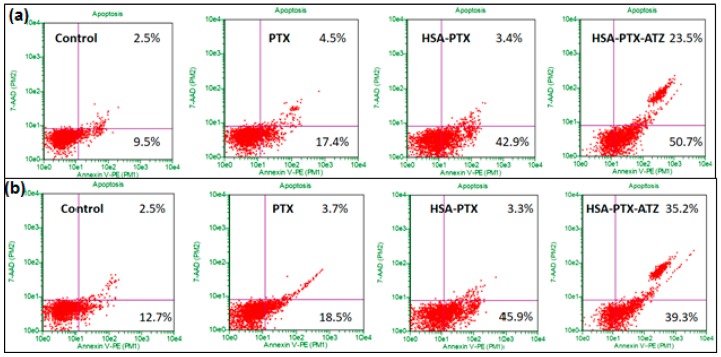
Apoptosis assay studies in (**a**) MDA-MB-231 and (**b**) MDA-MB-468 for the hypoxia targeting drug delivery system HSA-PTX-ATZ (The results are presented in terms of percentage of apoptotic cells similar to the previous literature [5,6,23,24,28,121]).

**Figure 8:**
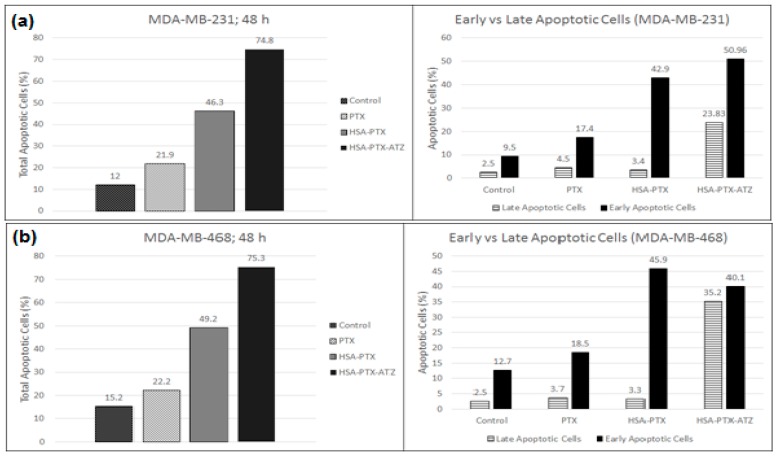
(**a**) Graphical representation of the apoptosis assay studies in MDA-MB-231; (**b**) Graphical representation of the apoptosis assay studies in MDA-MB-468 for the hypoxia targeting drug delivery system HSA-PTX-ATZ.

Apoptosis is induced in both the cell lines because of the treatment with the formulations as was analyzed by flow cytometry with Annexin V/7-AAD dual staining [20,120]. The levels of Annexin V+/7-AAD+ (R3), Annexin V−/7-AAD+ (R4), Annexin V−/7-AAD− (R5), and Annexin V+/7-AAD− (R6) were utilized to illustrate the percentage of live cells, early apoptotic, late apoptotic and necrotic cells. The percentage of apoptotic cells was observed to be significantly higher in both the cell lines treated with the targeted formulation when compared to free drug non-targeted formulations. The percentage of early and late apoptotic cells in the MDA-MB-231 cell line was observed to be around 50.7% and 23.5%, respectively, in targeted formulation treated cells and 42.9% and 3.4%, respectively, in the non-targeted formulation-treated cells (Figure 7a). The percentage of early and late apoptotic cells in MDA-MB-468 cells was observed to be around 39.3% and 35.2%, respectively, in targeted formulation treated cells and 45.9% and 3.3%, respectively, in the non-targeted formulation-treated cells (Figure 7b). The outcomes present a superior apoptosis-inducing capacity of the targeted formulation. The graphical representation of these results has been demonstrated in Figure 8a,b. These results were consistent with cytotoxicity studies, cell uptake studies and the confocal microscopic studies. Additionally, the blank formulation displayed generally low apoptosis in the cell line, demonstrating the safety of the carrier system when utilized for treatment.

## 3. Discussion

It is the objective of each targeted drug delivery system to develop a framework for the nano-delivery formulations to address the difficulties discussed so far by building up a reagent-free click chemistry process. There have been a few studies conducted and strategies created to set up such click chemical reactions as of late that are anything but difficult to be performed, are brisk and yield high amounts of these products with non-poisonous results. A few analysts now consider that click chemistry is a carefully engineered approach towards the development of new molecular delivery systems. This unique yet simple technique depends basically upon the development of carbon heteroatom bonds utilizing spring-loaded reactants. The increasing extent of utilization of these reactions in delivery systems is found in about all specializations of current pharmaceutical sciences from drug delivery to material sciences [121]. These sorts of click chemistry reactions extensively incorporate cycloaddition of unsaturated species: 1,3-dipolar cycloaddition, cycloaddition of unsaturated species: [4+2]-cycloaddition (Diels-Alder), nucleophilic substitution/ring-opening responses, carbonyl responses of the non-aldol sort, expansion to carbon-carbon numerous securities [122]. Because of the undeniable favorable circumstances of such simple chemical reactions, the click chemistry has been picking up in drug discovery, drug delivery, and bioconjugation reactions [40]. Its application is utilized as a strategy for creating libraries of such targeted drug delivery systems.

The formulation thus synthesized is a result of such a simple chemical process involving three steps only, thus creating a feasibility of formulating such delivery systems for a combination of ligands and carriers for several hydrophobic drugs of different categories and not just limited to the anti-cancer drugs. The safety of the carrier system thus developed is the key for the success of this formulation. Both the targeted and non-targeted formulations were prepared so that they can be compared to each other for their anti-cancer efficiency. The drug loading is suspected to be less in the formulation due to the long duration of the reaction and due to the size of the carrier molecule. However, the same spatial reasons can be given for the prolonged and sustained release of the drug into the tumor microenvironment.

The nanoparticles thus formed are found to be in the nanoscale of the particle size and the TEM results further confirm these results. The PDI was found to be quite low indicating that the size distribution is quite narrow in the particles which means that most of the nanoparticles were about the same size as the average particle size. The particles being in the nano size also promote the entry of the formulation into the tumor cells through the leaky vasculature by the EPR effect [61,88]. Furthermore, the particle size below 300 nm also helps prevent opsonization of the proteins via macrophages in the body that untimely leads to elimination of the formulation from the targeted tumor site. The results of the DLS and TEM are corroborative and form the basis for the results of the cytotoxicity studies.

The formulation further tested for the stability shows that the nature of the formulation is maintained close to the nature at the point of its synthesis. Thus, the formulation is found to be robust with little drug loss. This is an indication towards the long shelf-life possible by this formulation strategy. The storage conditions may also well be described from the results of this study as being stored at freezing temperatures for the longest shelf-life with the best possible stability and minimal drug loss during storage.

The cytotoxic studies were then performed to check the efficiency of the formulation in vitro. The formulation was found to have a little bit more efficacy in the MDA-MB-468 cell lines which have an overexpression of the extracellular CA IX receptor under hypoxic conditions and, hence, show good response to treatment. This effect is more or less the same in MDA-MB-231 cells [123]. Hence, the formulation does show a reasonable efficiency in this cell line also showing that it is a promising strategy to treat this more aggressive cell line [124]. Hence, based on the literature [124], we established a proof of concept that this formulation is effective in hypoxic conditions equally in both cell lines. The hypoxic conditions are found to have more of an effect because of the lowering of the pH in the swiftly proliferating cancer cells that results in the overexpression of this receptor and, thus, the formulation being taken up more effectively. The difference in cell viability between the targeted and non-targeted formulations is an indication of the fact that the formulation is being taken up not only by EPR effect but also by the targeted entry via the extracellular CA IX receptor; i.e., receptor mediated cell uptake [14]. The high cell viability in the blank formulation is proof of the safety of the carrier-ligand system. Furthermore, the difference in the response to this formulation in the normoxic (higher cell viability) and hypoxic conditions (lower cell viability) has led us to demonstrate that the uptake mechanism was CA IX receptor-mediated in TNBC cells. These studies establish the proof of concept of concept to show that this formulation is an efficient way to deliver drugs via targeting the hypoxia marker, CA IX. 

The in vitro cytotoxicity studies were followed up by fluorescence spectroscopic studies that illustrated the extent of uptake of the formulation in the cells. The comparative results between the non-targeted and targeted formulations show that the targeted formulation has a more preferential uptake in hypoxic conditions and the results show that the uptake is time-dependent. Thus, it corroborates the results of the cytotoxicity studies showing receptor-mediated uptake [13,31,106,125,126,127] of the formulation in addition to the EPR effect. The uptake of the non-targeted formulation may be explained as being purely a response to the EPR effect. Acetazolamide is a very established ligand of CA IX [112,128,129,130,131,132,133]. Our data of high cell killing effects of HSA-PTX-ATZ in hypoxic condition compared to normoxia and higher uptake of Rhodamine-labelled HSA-PTX-ATZ indicate the CA IX-mediated drug delivery effect of HSA-PTX-ATZ.

The findings of the cytotoxic studies and the fluorescence spectroscopic studies are further strengthened by the percentage of early and late apoptotic cells in both cell lines. The higher percentage of apoptotic cells proves the efficiency of the targeted formulation in both cell lines; this demonstrates the advantage of the targeting ligand. The low percentage of total apoptotic cells in the cell with the carrier system shows that the system is safe for normal cells.

## 4. Materials and Methods

### 4.1. Synthesis of the Hypoxia Targeting Drug Delivery System

**Figure 9:**
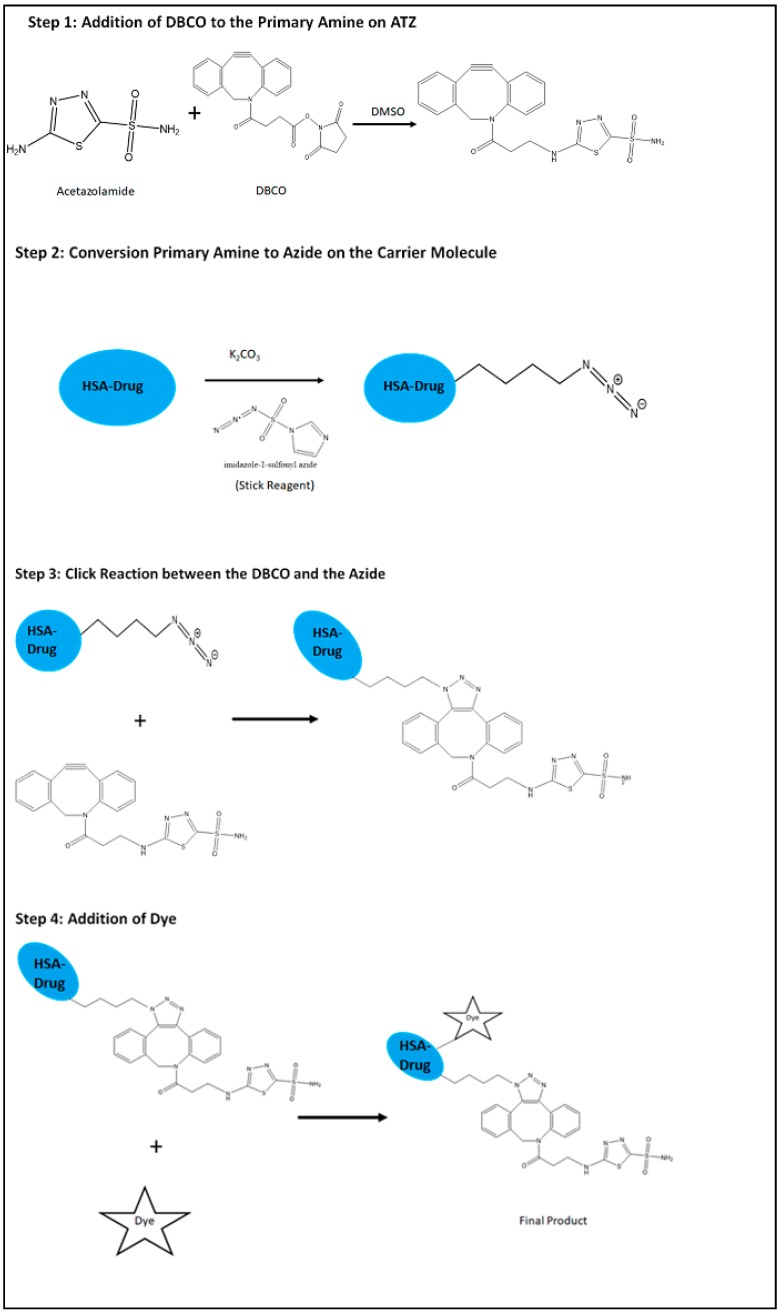
Schematic representation of the synthesis of the hypoxia targeting drug delivery system HSA-PTX-ATZ.

The synthesis of the drug delivery system developed in this study that targets hypoxia in the tumor cells is a simple four step utilizing click chemistry. As mentioned earlier, click chemistry is a quick process of conjugating two molecules that have complimentary functional groups that just ‘*click*’ with each other in minimal conditions of the reaction. The specific form of click reaction used in this study is the copper free cyclic Alkyne-Azide click reaction. The convenience of this reaction process allows the development of a library of ligands and carrier molecules that have these complimentary molecules which can take part in click reaction that can be made on a need-basis. The scheme of this reaction is presented in the Figure 9.

The description of this process is as follows:

#### 4.1.1. Preparation of the Targeting Ligand

The first step is to prepare the ligand on the HSA molecule. The starting material is the Acetazolamide. The amide group of the acetazolamide is first converted to primary amine by acid hydrolysis using 1 M HCl. The primary amine group thus activated is then used to be attached to one of the complimentary molecule, DBCO with a ––C≡≡C–– group that further participates in the click reaction. The acetazolamide with the primary amine group and the DBCO are dissolved in DMSO and then allowed to react for overnight at room temperature while stirring continuously. The product is then dialyzed in a 12 kD dialysis bag (Spectrapor, Spectrum Research facilities, SD) for 4–8 h and kept ready for further conjugation. 

#### 4.1.2. Preparation of the Carrier Molecule

Preparation of the carrier molecule is the next step. This involves conversion of the amine group of the amino acids comprised in the HSA protein to azide group. This involves two steps. First, the amine groups are converted to azide groups, ^−^N== ^+^N== ^−^N , using the Stick Reagent (imidazole-1-sulfonyl azide) in the presence of K_2_CO_3_ overnight at room temperature and continuous stirring. The addition of these two components of the reaction is carried out in ice because they are highly exothermic reactions. After the conversion, the azide product is dialyzed in a 12 kD dialysis bag (Spectrapor, Spectrum Research facilities, SD) for 4–8 h and the drug is loaded onto it.

#### 4.1.3. Drug Loading on the Carrier Molecule Comprising the Azide Group

The drug loading is performed by desolvation method that is derived from the coacervation process using previously described method in the literature [28,69,134,135]. Paclitaxel is a hydrophobic drug that is dissolved in ethanol. HSA was at first completely dissolved in phosphate buffer made in deionized water (50 mg/mL) and placed on a stirrer at a speed of around 600 rpm. The pH of the buffer is maintained at 8. The ethanolic solution of the PTX is then added to the HSA solution at the rate of 1 mL/min. The pH is again maintained at 8 using the same buffer. After half an hour of stirring, 8% glutaraldehyde is added to the reaction mixture to promote crosslinking in the HSA for encapsulating the drug effectively. The reaction is continued overnight while stirring at room temperature. The product is then dialyzed in a 12 kD dialysis bag (Spectrapor, Spectrum Research facilities, SD) for 4–8 h. This drug loaded carrier molecule with the azide group contributes the other complimentary molecule of the click reaction.

#### 4.1.4. Conjugation of the Ligand to the Carrier Molecule

This is the final step of the process of synthesis of the hypoxia targeting drug delivery system. It involves the conjugation of the alkyne group on DBCO and the azide group on the carrier molecule click conjugate with each other. This is the simplest step of the process where in the ligand containing the DBCO and the carrier molecule with the azide are combined together in a reaction under pH condition of 8 and at room temperature while continuously stirring at 600 rpm. The reaction is carried out for 4–6 h. The final product is dialyzed in a 12 kD dialysis bag (Spectrapor, Spectrum Research facilities, SD) for 2 h and lyophilized. The final product is water soluble. The product can also be attached further with an NIR dye by the similar click reaction to produce a theranostic product. This is further taken up for characterization studies using FTIR and NMR studies to confirm the reaction. 

### 4.2. Drug Loading

The process of drug loading was performed at room temperature as described earlier in the synthesis section. The calculation of percentage of drug loading was performed in HPLC and UV Spectrophotometer and the amount of drug encapsulated was found out using the standard graph previously developed by the methods specified in literature for PTX in both the HPLC and UV Spectrophotometer. A series of dilutions were made for the pure PTX and the absorbance for each was taken at 227 nm. A standard graph was plotted according to the readings. Later, a specified amount of the product has been taken and tested for absorbance. The amount of drug encapsulated from the line equation obtained from the standard graph.

### 4.3. Particle Size Analysis

The nanoparticles were further taken for particle size studies using a Beckman Coulter Delsa Nano-C DLS Particle analyzer (Beckman Coulter, Inc., Fullerton, CA, USA) that involved a 658 nm He–Ne laser as reported earlier by our lab. Tests were also examined by JEOL Transmission Electron Magnifying Instrument outfitted with LaB6 filament gun (JEM 2010, Tokyo, Japan) at an accelerating voltage of 200 kV for studying the particle morphology [6,8,53]. The nanoparticles were also depicted for surface morphology by Transmission Electron Microscopy (TEM). Tests were set up as described in the previous literature. Determined measure of each sample (4 µL) was applied to a Formvar-coated, carbon stabilized copper matrix (400 work). The copper matrix was air-dried, stained negatively with 5% aqueous uranyl acetate, and allowed to dry. 

### 4.4. Drug Release Studies

Drug release studies were performed with the formulation to assess the extent to which the drug has been released out from the formulation. Ideally, this is done in the physiological conditions. For this study, the studies were carried out at pH 7. The analysis was carried out at room temperature. A fixed volume of the formulation was placed in a dialysis bag under sink condition and continuous stirring. Specific volumes of samples were extracted at 2, 4, 8, 10, 12, 24, 48, 72 h from the dialysis bag. The nanoparticles were subjected to high shear in a sonicator for about 15–30 min to disrupt the nanoparticle formation so that the entire drug is released out and then the mixture was centrifuged at high speed of 15,000 rpm for 15 min. This forms a pellet of the disrupted HSA (being a heavy molecule in comparison to the drug) at the bottom and the supernatant contains the drug. The supernatant was extracted, diluted and then analyzed for drug content in UV and HPLC. The amount of was calculated from the line equation obtained from the standard graph and subtracted from the amount in the sample first taken to get the amount of drug released.

### 4.5. Stability Studies

The stability studies were performed to check for the stability of the formulation over a period of time in terms of particle size, polydispersity index (PDI), and drug loss. The formulation prepared was stored at three different conditions of temperatures, i.e., at room temperature (25 °C), under refrigeration (4 °C), and under frozen conditions (−20 °C) for 12 weeks [5,98]. The samples were retrieved every week and analyzed for the particle size, and PDI in DLS and drug content was analyzed in HPLC and UV.

### 4.6. Cell Culture

The cell lines chosen for this study are the MDA-MB-231 and MDA-MB-468 both of which correspond to the Triple Negative Breast Cancer (TNBC) [136,137]. These were the choice of cell lines because they are known to express the hypoxia marker Carbonic Anhydrase IX (CA IX) receptor on the surface of the cell lines. The MDA-MB-468 is found have a higher expression of the receptor compared to the MDA-MB-231 according to literature. The MDA-MB-231 is also found to be a more aggressive cell line that presents a greater difficulty for treatment. MDA-MB-231 is a stellate shaped cell and the MDA-MB-468 is a grape-like cluster of cells. Both the cell lines were cultured in Dulbecco’s Modified Eagle’s Medium (DMEM; Fisher Scientific, Waltham, MA, USA). The media was added with 10% fetal bovine serum (FBS) and streptomycin sulfate (10 mg/L). All cell lines were incubated at 37 °C in a 5% CO_2_ air humidified atmosphere. 

### 4.7. In Vitro Cytotoxicity Studies

The in vitro cytotoxicity studies were performed after the cells were induced with hypoxia using CoCl_2_. This treatment enhances the overexpression of the CA IX receptor on the surface of the cells. The assay was performed using the MTT reagent solution in PBS (1 mg/mL) at pH 7.4. The treatments included free drug (positive control), free carrier (negative control), carrier-drug (positive control), carrier-ligand (negative control), and the formulation comprising carrier-ligand-drug. The cells were seeded in 96-well plates with a normal of 5000 cells in each well. After incubating these cells for 24 h, they were treated with different concentrations of the formulations within a range of 0.25–5 µm. The treated cells were further incubated in the presence of the formulations for 48 h at 37 °C, after which the MTT reagent solution was added. The cells were incubated furthermore at 37 °C for 2 h. Following this, the media was supplanted by DMSO and the plates were put on a shaker for 10 min. The absorbance was measured at 590 nm utilizing a high-performance multi-mode plate reader (Synergy 2, BioTek, Winooski, VT, USA). The extent of surviving cells was calculated in terms of percentage by contrasting the absorbance of the treated cells and proper controls cells. This was performed on both the cell lines individually. Statistical analysis was done to calculate the significance of difference between the responses to the formulation in both cell lines.

### 4.8. Comparative In Vitro Cytotoxicity Studies for Normoxic and Hypoxic Conditions

Healthy cells and cancer cells show a difference in terms of development of hypoxia and acidic conditions in the microenvironment. The healthy cells do not overexpress the CA IX receptor and do not exhibit hypoxia while the cancer cells do. Thus, this study was performed to understand the difference in the uptake of specifically the formulation in the normoxic conditions as well as the hypoxic conditions in the said cell lines. This study is believed to give an assessment of the effect of the formulation on the normal and the tumor cells. The study was done similar to the in vitro cytotoxicity studies described above in two groups of treatments; the cells that are treated with CoCl_2_ to induce hypoxia and CA IX expression and the cells that are not treated with CoCl_2_ for normoxia. The assay was performed using MTT reagent solution (1 mg/mL) in both the cell lines. The assay was performed at the IC_50_ concentration of specifically the formulation to prove its efficiency in the hypoxic conditions comparatively.

### 4.9. Fluorescence Spectroscopic Studies

This assay is a quantitative measure of the amount of formulation encapsulating the drug entering into the cells using fluorescence spectroscopy. Both the targeted and non-targeted formulations were conjugated with Rhodamine B according to a previously established method. The cell-lines MDA-MB-231 and MDA-MB-468 were treated with CoCl_2_ first, then cultured in two separate 6 well-plates with each well comprising of about 100,000 cells in 2 mL of the media. These cells were then treated with the rhodamine conjugated formulations at their IC_50_ concentrations and incubated. Samples were collected every 4 h from the start of the treatment. Each sample collection procedure consisted of removing the formulation, washing it with PBS, and finally streaking the cells to collect the proteins and then further protein collection using lysis buffer. During the measurement of florescence, a control was also used that contained a mixture of methanol and the lysis buffer. 

### 4.10. Apoptosis Assay by Flow Cytometry

Apoptosis assay was performed on the MDA-MB-231 and MDA-MB-468 cell lines separately according to the prior literature. The cells were induced with hypoxia by treating them with CoCl_2_, then the cells were cultured in 6-well plates at the rate of 100,000 cells per well. The cells were incubated for 24 h at 37 °C under 5% CO_2_, followed by the treatment of the cells with free drug, non-targeted formulation and the targeted formulation and incubating further for 48 h to induce apoptosis. The concentrations of the formulations were the IC_50_ concentrations of the respective formulations obtained from the in vitro cytotoxicity assay. After a 48-h incubation, the cells were gathered, and the test was set up as indicated by the procedure described for Guava Nexin Annexin V assay (EMD Millipore, Billerica, MA, USA). Then, the media before trypsinization and the treated trypsinized cells were gathered in 15 mL tubes for each sample and centrifuged at 800 for 5 min. Cell pellets formed thus were dispersed in PBS at pH 7.4 containing 1% FBS so that the quantity of cells of the order of 2 × 10^5^ to 1 × 10^6^ cells/mL. 100 μL of these cell dispersions of each sample was mixed with 100 μL of the Guava Nexin Reagent and was incubated for 20 min at room temperature in the absence of sunlight. The resultant final samples were analyzed by Guava Easycyte Flow Cytometer (EMD Millipore).

## 5. Conclusions

Hypoxia is a target that is ubiquitously present in almost all cancers and is mostly found in the oxygen-deprived core of the tumor. It is a challenge to target it because of its location. However, the fast-growing cancer cells produce a lack of oxygen and a lowering of pH in the cancer cells which result in the overexpression of the surface receptor CA IX. This is a convenient cancer cell marker that allows the penetration of the nanocarrier to the core and, thus, effectively delivers the drug to the targeted site. This study is an attempt to establish the proof-of-concept that this can be achieved via the use of CA IX-targeting ligands like Acetazolamide and a bio-safe carrier like the human serum albumin which is abundant in the body and, hence, is non-immunogenic. This study succeeded in proving the usefulness of the formulation synthesized herein and characterized further. It shows that the cells selectively take up the formulation and shows a low cell viability in cancer cells with more percentage of apoptotic or dying cells. The results also show that the carrier system is safe and hence can be used for drug delivery. The delivery system, therefore, helps in the delivery of drugs to specific sites; thus, eliminating the undesired side effects on healthy cells. The nature of the formulation further allows the conjugation of a dye that can potentially make it a theranostic system. Thus, it can be safely said that the formulation can be further used for in vivo evaluations that are currently underway in our laboratory.

## Supplementary Materials

Supplementary materials can be found at http://www.mdpi.com/1422-0067/19/3/838/s1.
