# Supplementary material for: Copper-Free ‘Click’ Chemistry-Based Synthesis and Characterization of Carbonic Anhydrase-IX Anchored Albumin-Paclitaxel Nanoparticles for Targeting Tumor Hypoxia
Source: Int J Mol Sci. 2018 Mar 13;19(3):838. doi: 10.3390/ijms19030838 (PMC5877699; doi:10.3390/ijms19030838)
Supplement: Supplementary file 1 [file ijms-19-00838-s001.pdf]

Supplementary Materials

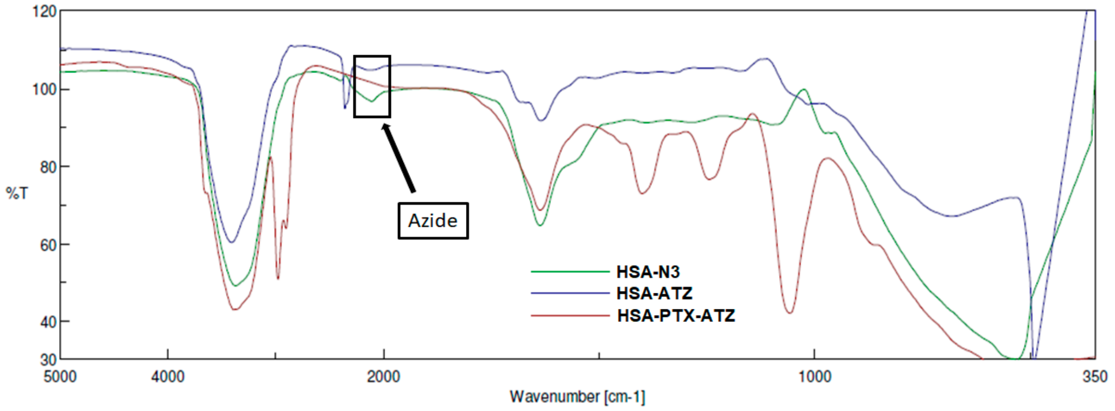

Figure S1. FTIR for the hypoxia targeting drug delivery system HSA-PTX-ATZ.

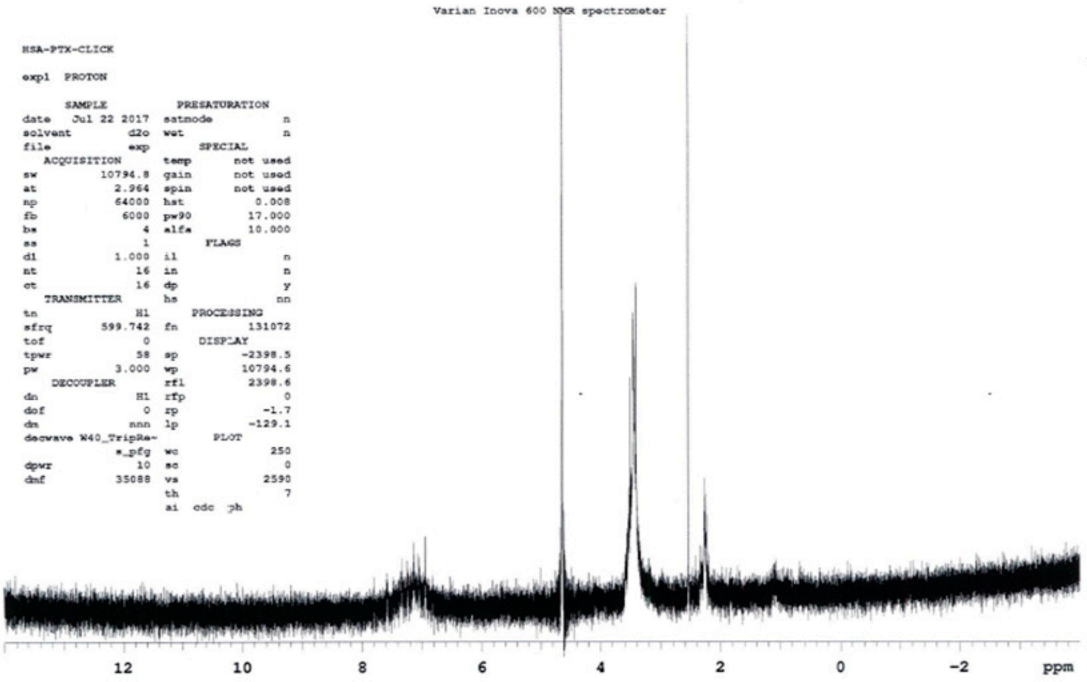

Figure S2. NMR spectrum for the hypoxia targeting drug delivery system HSA-PTX-ATZ.
